# The catalogue of *Mycobacterium tuberculosis* mutations associated with drug resistance to 12 drugs in China from a nationwide survey: a genomic analysis

**DOI:** 10.1016/S2666-5247(24)00131-9

**Published:** 2024-11

**Authors:** Shaojun Pei, Zexuan Song, Wei Yang, Wencong He, Xichao Ou, Bing Zhao, Ping He, Yang Zhou, Hui Xia, Shengfen Wang, Zhongwei Jia, Timothy M Walker, Yanlin Zhao

**Affiliations:** a Department of Global Health, School of Public Health, Peking University, Beijing, China; b National Center for Tuberculosis Control and Prevention, Chinese Center for Disease Control and Prevention, Beijing, China; c National Key Laboratory of Intelligent Tracking and Forecasting for Infectious Diseases, Chinese Center for Disease Control and Prevention, Beijing, China; d National Clinical Research Center for Infectious Diseases, Shenzhen Third People’s Hospital, Shenzhen, China; e Oxford University Clinical Research Unit, Ho Chi Minh City, Viet Nam; f Nuffield Department of Medicine, Centre for Tropical Medicine and Global Health, University of Oxford, Oxford, UK

## Abstract

**Background:**

WHO issued the first edition catalogue of *Mycobacterium tuberculosis* complex (MTBC) mutations associated with drug resistance in 2021. However, country-specific issues might lead to arising complex and additional drug-resistant mutations. We aimed to fully reflect the characteristics of drug resistance mutations in China.

**Methods:**

We analysed MTBC isolates from the nationwide drug-resistant tuberculosis surveillance with 70 counties in 31 provinces, municipalities, and autonomous regions in China. Three types of MYCOTB plates were used to perform drug susceptibility testing for 12 antibiotics (rifampicin, isoniazid, ethambutol, levofloxacin, moxifloxacin, amikacin, kanamycin, ethionamide, clofazimine, linezolid, delamanid, and bedaquiline). Mutations were divided into five groups according to their odds ratios, positive predictive values, false discovery rate-corrected p values, and 95% CIs: (1) associated with resistance; (2) associated with resistance—interim; (3) uncertain significance; (4) not associated with resistance—interim; and (5) not associated with resistance. The Wilcoxon rank-sum and Kruskal–Wallis tests were used to quantify the association between mutations and minimum inhibitory concentrations (MICs). Our dataset was compared with the first edition of the WHO catalogue.

**Findings:**

We analysed 10 146 MTBC isolates, of which 9071 (89·4%) isolates were included in the final analysis. 744 (8·2%) isolates were resistant to rifampicin and 1339 (14·8%) to isoniazid. 208 (1·9%) of 11 065 mutations were classified as associated with resistance or associated with resistance—interim. 33 (97·1%) of 34 mutations in group 1 and 92 (52·9%) of 174 in group 2 also appeared in groups 1 or 2 of the WHO catalogue. Of 81 indel mutations in group 2, 15 (18·5%) were in the WHO catalogue. The newly discovered mutation *gyrA*_Ala288Asp was associated with levofloxacin resistance. MIC values for rifampicin, isoniazid, moxifloxacin, and levofloxacin corresponding to resistance mutations in group 1 were significantly different (p<0·0001), and 12 high-level resistance mutations were detected. 61 mutations in group 3 occurred as solo in at least five phenotypically susceptible isolates, but with MIC values moderately higher than other susceptible isolates. Among 945 phenotypically resistant but genotypically susceptible isolates, 433 (45·8%) were mutated for at least one efflux pump gene.

**Interpretation:**

Our analysis reflects the complexity of drug resistance mutations in China and suggests that indel mutations, efflux pump genes, protein structure, and MICs should be fully considered in the WHO catalogue, especially in countries with a high tuberculosis burden.

**Funding:**

National Key Research and Development Program of China and the Science and Technology Major Project of Tibetan Autonomous Region of China.

## Introduction

Tuberculosis is a chronic infectious disease caused by *Mycobacterium tuberculosis*, which is mainly treated with anti-tuberculosis drugs. Globally, tuberculosis remains a major public health threat and is the second biggest infectious disease killer after the COVID-19 pandemic. In 2021, an estimated 10·6 million (95% uncertainty interval [UI] 9·9–11·0) people worldwide had tuberculosis.^1^ Furthermore, drug-resistant tuberculosis is a persistent threat. Globally, there were an estimated 450 000 incident cases (95% UI 399 000–501 000) of multidrug-resistant (MDR) or rifampicin-resistant tuberculosis in 2021, and the estimated proportion of MDR or rifampicin-resistant tuberculosis cases with pre-extensively drug-resistant (pre-XDR) tuberculosis was 20% (95% CI 16–26).^1^ However, the number of people who were detected with MDR or rifampicin-resistant tuberculosis and received treatment fell short of the estimated incident cases of MDR or rifampicin-resistant tuberculosis each year.^1^ The COVID-19 pandemic aggravated an already suboptimal global tuberculosis response.^2^

**Research in context**

**Evidence before this study**

We searched PubMed for articles published in English between database inception and March 10, 2023, using the search terms "tuberculosis", “drug resistance”, "mutation", "China" (interchanged with "Chinese"), and "catalogue" (interchanged with "database" or "compendium"). We found 69 studies including 26 based on isolates collated from some provinces or regions in China, 32 involving first-line and second-line drugs or new and repurposed drugs, and 11 with scarce data. We based our methods on the 2021 first edition of the WHO catalogue on *Mycobacterium tuberculosis* complex mutations associated with drug resistance (by the CRyPTIC Consortium and the Seq&Treat Consortium). China is one of the 30 countries that had a severe multidrug-resistant or rifampicin-resistant tuberculosis epidemic. The misuse of antibiotics has further led to the complex and diverse drug-resistant profiles in China.

**Added value of this study**

To our knowledge, this study included the largest phenotypic and genotypic matched dataset for *M tuberculosis* in China to date, with 70 counties randomly selected by nationwide drug-resistant tuberculosis surveillance and good representation across the country. The entire analysis process was based on approaches used in the WHO catalogue. This study validates the WHO catalogue and reflects the characteristics of drug resistance mutations in China. The results suggest that indel mutations, efflux pump genes, protein structure, and minimum inhibitory concentrations should be fully considered in the WHO catalogue, especially in countries with a high tuberculosis burden.

**Implications of all the available evidence**

WHO generated a catalogue of mutations in 2021 to establish a global standard for genotypic resistance prediction of *M tuberculosis*. However, the number of isolates sampled in some countries might not have been sufficient to fully reflect the distribution of resistance mutations. Our study shows the feasibility of applying the WHO catalogue to existing data from routine work or country-wide surveillance.

Achieving 5-year global tuberculosis targets will require optimised use of existing and new tools of detection and treatment. For people to receive the correct diagnosis and begin the most effective treatment regimen as early as possible, the WHO End TB Strategy calls for universal access to drug susceptibility testing.^1^^,^^3^ However, phenotypic drug susceptibility testing for *M tuberculosis* typically takes more than a month to complete and requires expensive and complex laboratory capacity, and has high requirements for biosecurity protection.^4^ In the past 20 years, genotypic approaches such as nucleic-acid amplification tests and whole-genome sequencing (WGS)—which are highly specific and sensitive—have helped to revolutionise the tuberculosis diagnostic landscape.^5^^,^^6^ To establish a global standard for genotypic resistance prediction, WHO generated the first edition catalogue of mutations in 2021.^7^ In this catalogue, WHO analysed 41 137 *Mycobacterium tuberculosis* complex (MTBC) isolates with drug susceptibility testing and WGS data from 45 countries. The final mutation associations were stratified into five groups: (1) associated with resistance; (2) associated with resistance—interim; (3) uncertain significance; (4) not associated with resistance—interim; and (5) not associated with resistance. 1149 mutations were classified as associated with phenotypic resistance (groups 1 and 2) for 13 WHO endorsed anti-tuberculosis drugs.^7^ However, mutation sites of tuberculosis drug resistance vary between different countries and regions,^8^ and the number of isolates for each country in the WHO dataset was not sufficient to contain all representative resistance mutations. Undernutrition, poor housing, socially driven substance abuse, and poor access to health services, particularly in high tuberculosis burden settings might contribute to the increasingly complex drug-resistant profile.^2^^,^^9^

Among the 30 countries with high tuberculosis burden, there were an estimated 780 000 incident cases (95% UI 665 000–905 000) in China in 2021, which was lower than Indonesia and India. China is one of the 30 countries that had a severe MDR or rifampicin-resistant tuberculosis epidemic accounting for 7·3% of the global MDR tuberculosis burden in 2021.^1^ The misuse of antibiotics has further led to the complex and diverse drug-resistant profiles in China.^10^ For example, quinolone antibiotics have been widely used in the treatment of respiratory, gastrointestinal, and urinary tract bacterial infections in China during the past few decades; therefore, a high resistance rate of quinolones in China is mostly attributed to easy accessibility and misuse of these drugs for other infections.^11^ We aimed to fully reflect the characteristics of drug resistance mutations in China by generating a catalogue of MTBC drug resistance mutations from isolates based on approaches used in the first edition of the 2021 WHO catalogue.

## Methods

### Data sources

We analysed MTBC isolates from the nationwide drug-resistant tuberculosis surveillance, conducted by the Chinese Center for Disease Control and Prevention (CDC) using cluster randomised sampling, based on the first national survey of drug-resistant tuberculosis in China.^12^ 70 counties were selected in proportion with the number of reported smear-positive cases in 31 provinces, municipalities, and autonomous regions in China, and at least one county in each province-level unit was included. This study was approved by the Tuberculosis Research Ethics Review Committee of the Chinese CDC. Written informed consent was obtained from all participants.

### Phenotypic and genotypic data

Three types of MYCOTB plates (Thermo Fisher Scientific, Waltham, MA, USA) were used in this study (MYCOTBI, UKMYC5, and UKMYC6) to perform drug susceptibility testing for 12 antibiotics (rifampicin, isoniazid, ethambutol, levofloxacin, moxifloxacin, amikacin, kanamycin, ethionamide, clofazimine, linezolid, delamanid, and bedaquiline) and clofazimine, linezolid, delamanid, and bedaquiline were considered new and repurposed drugs (NRDs).^13^^,^^14^ The minimum inhibitory concentrations (MICs) of levofloxacin for MYCOTB were calculated as those of ofloxacin divided by 2. MICs for each drug were read by two trained operators (ZS and WH) using a Vizion Digital viewing system (Thermo Fisher Scientific) after incubation of plates for 10–21 days. Inconsistent results were reread by a third experienced operator (BZ). The proficiency test for MICs and phenotypic testing were qualified by the supernational reference laboratory (Antwerp, Belgium). Genomic DNA was extracted using the cetyltrimethylammonium bromide method and sequenced using the Illumina Hiseq 2000 platform (Illumina, San Diego, CA, USA). DNA variant calling and analysis were performed using the same pipeline (Github; version 0.8.3) as the one used for the 2021 first edition of the WHO catalogue (by the CRyPTIC Consortium and the Seq&Treat Consortium).^15^ An overview of the phenotypic and genotypic data processing is shown in appendix 2 (p 1).

### Statistical analysis

Phenotypic and genotypic data were analysed using MATLAB (version R2021b), RStudio (version 2021.09.1+372), and IBM SPSS Statistics Software (version 26). The critical concentration was used to translate MIC data into resistant or susceptible categories.^13^^,^^14^ Positive predictive values (PPVs) with binomial exact 95% CIs and odds ratios (ORs) with corresponding p values using Fisher's exact test were calculated via contingency tables generated from the number of susceptible and resistant phenotypes with and without each solo variant in the candidate genes.^15^ The candidate genes and critical concentration used for interpretation of the 12 anti-tuberculosis drugs are shown in appendix 2 (pp 1–2). Candidate genes were divided into two tiers according to their probability of containing resistance mutations.^15^ Tier 1 genes were considered those most probably to contain resistance mutations. Tier 2 included genes with a reasonable pretest probability of containing resistance. A Benjamini–Hochberg correction procedure was used to control for multiple testing, with a false discovery rate (FDR) of 5%. We further computed the proportion of mutations as the number of times the mutation was observed as a solo divided by the number of times the mutation was observed. The Wilcoxon rank-sum and Kruskal–Wallis tests were performed to further quantify the association between mutations and MICs. Two new indicators (the median of MIC and FDR-corrected significance of the Wilcoxon rank-sum test) were used to measure the relationship between the mutations and MIC values. We classified 31 provinces, municipalities, and autonomous regions in China into different parts by two regional division methods: one by classifying provinces into southern and northern parts according to their geographical location and the other by dividing provinces into eastern, central, and western parts according to their economic development. Then Pearson’s χ^2^ test was used to find the correlation between different regional parts and drug resistance mutations.

As per the WHO catalogue, the mutations were divided into five groups according to their ORs, PPVs, FDR-corrected p values, and 95% CIs: (1) associated with resistance; (2) associated with resistance—interim; (3) uncertain significance; (4) not associated with resistance—interim; and (5) not associated with resistance (ie, consistent with susceptibility). Any mutation with a PPV or PPV conditional on being solo of less than 10% with 95% confidence was considered neutral (groups 4 and 5; appendix 2 p 1).

Mutations were defined as groups 1 or 2 if they occurred in at least five isolates, had a 95% CI lower bound of at least 0·25 for the PPV, had an OR of at least 1, and a significant FDR-corrected p value. If these mutations also appeared in groups 1 or 2 of the WHO catalogue or had other literature evidence, they were defined as group 1 and the rest were group 2. Mutations that did not meet the above conditions and appeared in groups 1 or 2 of the WHO catalogue were defined as group 2 and the rest were group 3. Any non-synonymous mutation in the rifampicin-resistance determining region of *rpoB* and any premature stop codon, insertion, or deletion in *ethA* or *katG* was also interpreted as group 2 (appendix 2 p 1).

To investigate why *gyrA*_Ala288Asp triggers moxifloxacin or levofloxacin resistance, we modelled the *gyrA*_Ala288Asp using PyMOL (version 2.5) and compared with the wild-type *(gyrA*–*gyrB*)^dimer^–DNA–levofloxacin complex x-ray structure (PDB: 5BTG). The six missing residues (Ser431 to Gly436) in the flexible loop1 (Ala425 to Gly440) of *gyrB* from the crystal structure were fixed by Modeller (version 9.10).

### Role of the funding source

The funders of the study had no role in study design, data collection, data analysis, data interpretation, or writing of the report.

## Results

**Figure 1:**
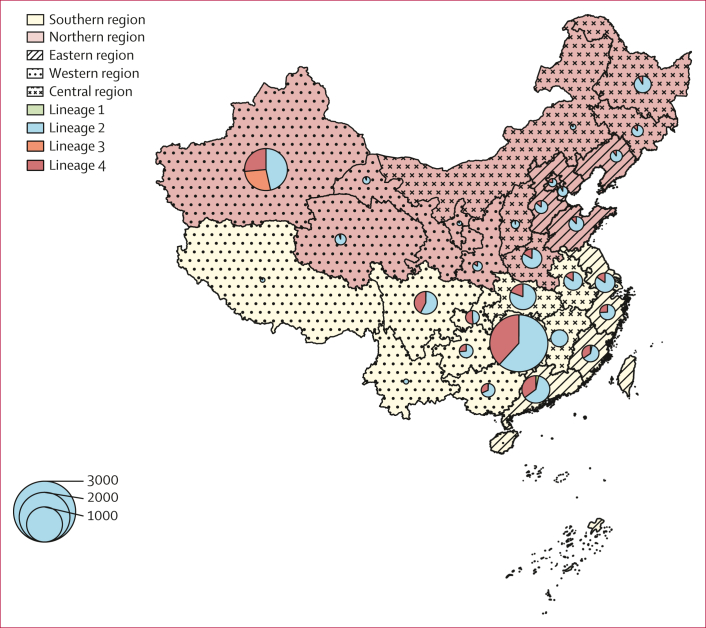
Distribution of *Mycobacterium tuberculosis* complex isolates in China used in this catalogue of genotype–phenotype associations Pie charts show the proportion of isolates among different lineages for each location. The size of pie charts corresponds with the number of isolates.

We analysed 10 146 MTBC isolates from surveillance of drug-resistant tuberculosis in China. 9071 (89·4%) isolates were included in the final analysis (appendix 3 p 1). Four main *M tuberculosis* lineages were observed in the dataset (figure 1). The largest number of isolates were contributed by Hunan province (2680 [29·5%]) and Xinjiang autonomous region (1426 [15·7%]). Most isolates (6127 [67·5%]) belonged to lineage 2, followed by 2526 (27·8%) in lineage 4, 386 (4·3%) in lineage 3, and 32 (<1%) in lineage 1. A strong correlation was found between the provinces where the isolates were collected and lineage (Pearson’s χ^2^ test; p<0·0001). Lineage 3 had the highest frequency in the Xinjiang autonomous region.

**Figure 2:**
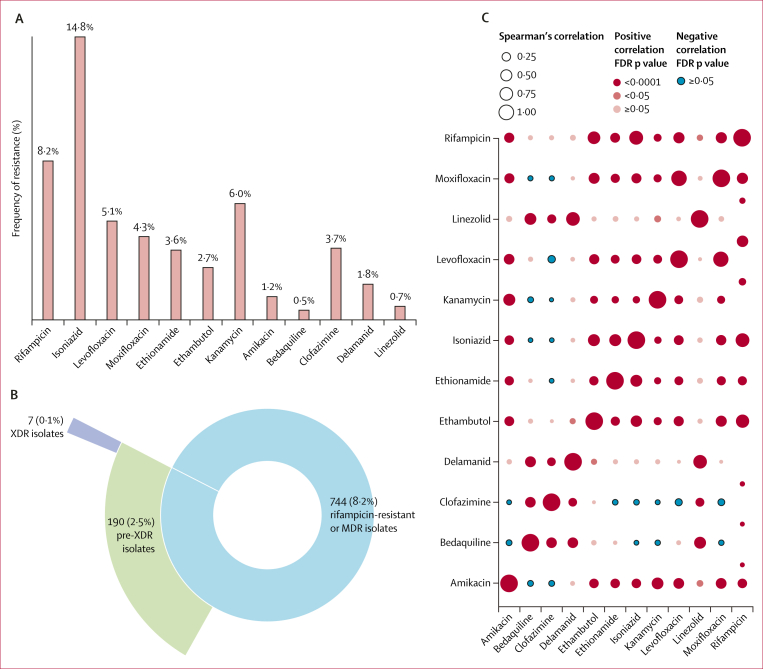
Drug resistance classification and correlation to 12 anti-tuberculosis drugs (A) The frequency of resistance to each of the 12 drugs. (B) Pie chart showing the distribution of MDR or rifampicin-resistant, pre-XDR, and XDR isolates. (C) Spearman’s correlation of resistance to 12 drugs. FDR=false discovery rate. MDR=multidrug resistant. pre-XDR=pre-extensively drug resistant.

The frequency of resistance to each of the 12 drugs is shown in figure 2A. 744 (8·2%) isolates were resistant to rifampicin and 1339 (14·8%) were resistant to isoniazid. A low proportion of isolates were resistant to NRDs bedaquiline (19 [0·5%]), clofazimine (134 [3·7%]), delamanid (66 [1·8%]), and linezolid (27 [0·7%]). According to new WHO definitions of pre-XDR and XDR, 190 (2·5%) isolates were further classified as pre-XDR and seven (0·1%) were XDR. Rifampicin resistance was the most strongly associated with resistance to each of the other drugs (Spearman’s correlation; p=0·047) except for bedaquiline, clofazimine, and delamanid (figure 2C). Apart from correlations between drugs in the same class, such as moxifloxacin and levofloxacin (fluoroquinolones), the correlation between rifampicin and isoniazid was the highest (Spearman’s correlation 0·55; p<0·0001), and linkage disequilibrium of *rpoB*_Ser450Leu and *katG*_Ser315Thr was also the highest (0·14; appendix 2 pp 3–5). Although there was no correlation between NRDs and first-line or second-line drug resistance, the correlation within NRDs was proportionally higher. For example, the correlation between delamanid and linezolid (Spearman’s correlation 0·54; p<0·0001) was second only to the correlation between rifampicin and isoniazid (appendix 3 p 2).

**Figure 3:**
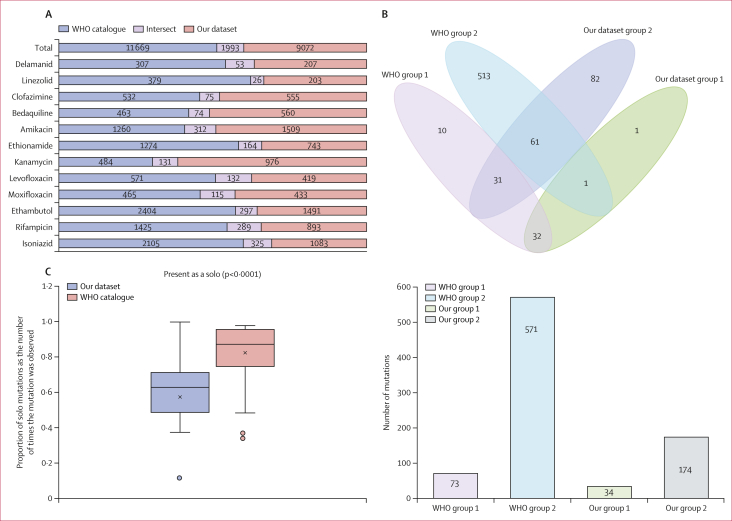
Comparison of the WHO catalogue and our dataset (A) The number of associations between the catalogues for each drug. (B) Venn diagram of the differences between our dataset and the first edition of the WHO catalogue for groups 1 and 2 mutations. (C) Boxplots of the proportion of solo mutations as the number of times the mutation was observed in groups 1 and 2 for our dataset and WHO data. The cross indicates the median, boxes indicate IQRs, bars indicate minimum and maximum values, and dots show outliers.

11 065 associations between mutations of the candidate genes and each of the 12 drugs were calculated, of which 1993 (18·0%) also appeared in the WHO catalogue (figure 3A). 208 (1·9%) of 11 065 mutations were classified as associated with phenotypic resistance (groups 1 or 2; appendix 4 pp 1–12). Most of these mutations were consistent with the WHO catalogue, and the difference with the WHO catalogue was mainly focused on insertion and deletion mutations. There were 34 mutations in group 1, of which 33 (97·1%) were in groups 1 or 2 of the WHO catalogue. 92 (52·9%) of 174 mutations in group 2 appeared in group 1 or 2 of the WHO catalogue (figure 3B). There were 81 indel mutations in group 2, but only 15 (18·5%) indel mutations were in the WHO catalogue (appendix 4 pp 1–5).

The present as a solo of mutations in our dataset was lower than that in the WHO catalogue (median 0·63 [IQR 0·50–0·71] *vs* 0·88 [0·77–0·95]; p<0·0001; figure 3C), further illustrating the complex and drug-resistant profile in China. All group 1 and 2 mutations were derived from tier 1 genes. The only newly discovered mutation in our dataset was *gyrA*_Ala288Asp, which was associated with levofloxacin resistance (appendix 2 pp 6–7). This mutation appeared as the solo mutation in five isolates, all of which were resistant to levofloxacin. The diagnostic values for drug resistance of mutations in group 1 are shown in appendix 2 (pp 6–7).

In our dataset, we found a new mutation associated with levofloxacin resistance *gyrA*_Ala288Asp, with results from the phylogenetic analysis (appendix 2 p 8) showing that isolates with *gyrA*_Ala288Asp were not a monophyletic group. To examine structural differences, we compared the modelled Ala288Asp mutant with the wild-type structure. In the wild-type, a huge pore can be seen around loop1 and Ala288Asp, linking the deeply buried levofloxacin with a tunnel; whereas in the carboxyl group on the Ala288Asp mutant, the long side chain of Asp288 of *gyrA* has reached loop1 and formed a salt bridge with the hydroxyl group of Ser431. This formation results in a narrower bottleneck around the pore of the tunnel in the Ala288Asp mutant (appendix 2 p 8). Given that the pore is possibly involved in drug entry, we extrapolate that the Ala288Asp mutation might obstruct the entrance with its extended side chain and hydrogen bonds to loop1, thereby preventing levofloxacin from reaching its binding domain.

Correlations were found between different Chinese regions and rifampicin-resistant and fluoroquinolone-resistant mutations (appendix 2 pp 9–13). Among rifampicin-resistant mutations, there were three mutations (*rpoB*_Ser450Leu, *rpoB*_His445Tyr, and *rpoB*_Ser450Trp) with differences in geographical distribution. The proportion of *rpoB*_His445Tyr in the eastern region was significantly higher than that in the middle and western regions of China (21 [19·8%] of 106 *vs* 30 [9·0%] of 333 *vs* 16 [11·9%] of 135; p=0·010), and the same finding was seen for *rpoB*_Ser450Trp (five [4·7%] *vs* six [1·8%] *vs* zero; p=0·029). The proportion of *rpoB*_Ser450Leu in the middle region was significantly higher than that in the eastern and western regions of China (222 [66·7%] *vs* 54 [50·9%] *vs* 81 [60·0%]; p=0·012). For fluoroquinolone-resistant mutations, there are differences in the geographical distribution of *gyrA*_Asp94Tyr, *gyrA*_Ala288Asp, and *gyrB*_Asp461Asn. Among these, the proportion of *gyrA*_Asp94Tyr in the northern region was significantly higher than that in the southern region of China (18 [17·3%] of 104 *vs* 13 [5·2%] of 249; p=0·020), and the proportion in the eastern region was significantly higher than that in the middle and west regions (16 [19·0%] of 84 *vs* 11 [5·1%] of 216 *vs* four [7·7%] of 52; p=0·0010; appendix 2 pp 11–13). Although there was no significant correlation between different northern and southern regions of China and isoniazid-resistant mutations (p=0·102), the proportion of *katG*_Ser315Thr in the southern region was numerically higher than that in the northern region for lineage 4 (98 [62·8%] of 156 *vs* 14 [40·0%] of 35; p=0·021; appendix 2 pp 9–11).

The MIC values for rifampicin, isoniazid, moxifloxacin, and levofloxacin corresponding to various resistance mutations in group 1 were significantly different (p<0·0001), and 12 high-level resistance mutations were detected (table 1). There were eight *rpoB* mutations associated with rifampicin resistance, of which three (Ser450Leu, His445Tyr, and Ser450Trp) were associated with higher MIC values (median >8·0), especially the *rpoB_*Ser450Trp mutation—although infrequent—was associated with high-level rifampicin resistance. For the four mutations related to isoniazid, the MIC values of *katG* mutations (Ser315Thr and Ser315Asn) were significantly higher than those of *inhA* mutations (−777C→T and −154G→A). For *gyrA* the median MIC values of Asp94Asn and Asp94Gly were higher than those of Asp94Tyr, Asp94His, and Asp94Ala for moxifloxacin and levofloxacin. Additionally, we found 61 mutations in group 3 that occurred as solo in at least five phenotypically susceptible isolates, but with MIC values moderately higher than other susceptible isolates (appendix 4 pp 1–12). For isoniazid, the MIC values of *katG*_Gly156Cys, *ndh*_Val18Ala, and *mshA*_Ala187Thr had increased to the critical concentration.

**Table 1:** Resistance levels for mutations in grade 1

	Minimum inhibitory concentrations (mg/L)	p value
Rifampicin (ref 0·5 mg/L)	..	<0·0001
*rpoB*_Ser450Leu	16·0 (8·0–16·0)∗	..
*rpoB*_His445Asp	8·0 (4·0–16·0)∗	..
*rpoB*_Leu452Pro	2·0 (1·0–4·0)	..
*rpoB*_Asp435Tyr	2·0 (2·0–4·0)	..
*rpoB*_His445Tyr	12·0 (4·0–16·0)∗	..
*rpoB*_Asp435Val	8·0 (4·0–16·0)∗	..
*rpoB*_His445Leu	4·0 (2·0–4·0)	..
*rpoB*_Ser450Trp	16·0 (16·0–16·0)∗	..
Moxifloxacin (ref 1·0 mg/L)	..	<0·0001
*gyrA*_Ala90Val	2·0 (1·0–2·0)	..
*gyrA*_Ser91Pro	4·0 (2·0–4·0)	..
*gyrA*_Asp94Gly	4·0 (4·0–8·0)	..
*gyrA*_Asp94Tyr	3·0 (2·0–4·0)	..
*gyrA*_Asp94His	4·0 (2·0–4·0)	..
*gyrA*_Asp94Ala	2·0 (1·0–2·0)	..
*gyrA*_Asp94Asn	8·0 (4·0–8·0)∗	..
Levofloxacin (ref 1·0 mg/L)	..	<0·0001
*gyrA*_Ala90Val	8·0 (4·0–8·0)∗	..
*gyrA*_Ser91Pro	4·0 (2·0–8·0)	..
*gyrA*_Asp94Gly	16·0 (8·0–16·0)∗	..
*gyrA*_Asp94Tyr	8·0 (4·0–16·0)∗	..
*gyrA*_Ala288Asp	2·0 (1·0–2·0)	..
*gyrA*_Asp94His	4·0 (4·0–8·0)	..
*gyrA*_Asp94Ala	4·0 (2·0–8·0)	..
*gyrA*_Asp94Asn	16·0 (8·0–16·0)∗	..
*gyrB*_Asp461Asn	4·0 (3·0–6·0)	..
Isoniazid (ref 0·1 mg/L)	..	<0·0001
*inhA*_−777C→T	0·3 (0·2–0·5)	..
*inhA*_−154G→A	0·2 (0·1–0·5)	..
*katG*_Ser315Thr	3·2 (1·6–4·0)∗	..
*katG*_Ser315Asn	2·0 (1·0–4·0)∗	..
Ethambutol (ref 4·0 mg/L)	..	0·38
*embB*_Met306Val	8·0 (8·0–8·0)	..
*embB*_Gln497Arg	8·0 (8·0–8·0)	..
*embB*_Tyr319Ser	8·0 (8·0–8·0)	..

Data are median (IQR), unless stated otherwise. Drug doses were the crucial concentration used for interpretation and the Kruskal–Wallis test was used to determine resistance levels. p values are comparing mutation-conferred minimum inhibitory concentrations with the reference standard.

∗ High-level drug resistance mutations.

The genetic-based predictions of resistance by groups 1 and 2 mutations and phenotypes derived from MICs were compared for all isolates in our dataset. Table 2 showed that ethambutol had the lowest PPV (54·7%), and 122 (77·2%) of 158 isolates with false positive prediction had MIC values of 4 mg/L corresponding to an area of technical uncertainty.^16^ For ethionamide, 61 (42·4%) of 144 isolates with false positive prediction were insertion and deletion mutations in the coding region of *ethA*, suggesting that *ethA* indels confer modest MIC increases, thereby supporting the need for a borderline category in addition to resistance and sensitivity.^13^ To investigate the reason for low sensitivity in all drugs mentioned in table 2, we analysed mutations in efflux pump genes of the phenotypically resistant isolates that are incorrectly identified as susceptible.^17^^,^^18^ Among 945 phenotypically resistant but genotypically susceptible isolates, 433 (45·8%) were mutated for at least one efflux pump gene and 71 (7·5%) had frameshift mutations in the glycerol kinase (*glpK*) gene, which might produce transiently heritable drug tolerance.^19^

**Table 2:** Genetic-based predictions of resistance by our catalogue

	True positives	False positives	True negatives	False negatives	PPV	NPV	Sensitivity	Specificity
Rifampicin	637	77	8248	107	89·2%	98·7%	85·7%	99·1%
Isoniazid	1026	87	7635	313	92·2%	96·1%	76·6%	98·9%
Levofloxacin	350	30	8576	114	92·1%	98·7%	75·4%	99·7%
Moxifloxacin	306	76	8600	88	80·1%	99·0%	77·7%	99·1%
Ethambutol	191	158	8673	52	54·7%	99·4%	78·6%	98·2%
Ethionamide	214	144	8601	111	59·8%	98·6%	65·8%	98·4%

True positives indicate the number of phenotypically resistant samples that are correctly identified as resistant. False positives indicate the number of phenotypically susceptible samples that are falsely identified as resistant. True negatives indicate the number of phenotypically susceptible samples that are correctly identified as susceptible. False negatives indicate the number of phenotypically resistant samples that are incorrectly identified as susceptible. PPV=positive predictive value. NPV=negative predictive value.

## Discussion

To our knowledge, this study included the largest phenotypic and genotypic matched dataset for *M tuberculosis* in China to date. The isolates were selected using nationwide surveillance of drug-resistant tuberculosis with good representativeness across China. Our analyses followed the processes used in the WHO catalogue, and MIC and phenotypic testing have passed proficiency testing by a supernational reference laboratory. Hence, this study comprehensively and accurately reflects the characteristics of drug resistance loci in China and might help to improve diagnosis and treatment of drug-resistant tuberculosis.

The frequency of resistance to the 12 drugs investigated in our dataset shows that the country has a serious problem of drug-resistant tuberculosis. 744 (8·2%) isolates were resistant to rifampicin and 1339 (14·8%) were resistant to isoniazid, which is slightly higher than shown in the 2007 national survey of drug-resistant tuberculosis in China,^12^ indicating that the situation of drug-resistant tuberculosis was still severe. However, our study did not include pyrazinamide, which was because of its preference for acidic conditions, as the drug cannot be successfully inoculated onto microtitre plates (such as MYCOTB).^20^ The low proportion of drug resistance to NRDs provides hope for the prevention and control of drug-resistant tuberculosis in China by treatment regimens that include NRDs. For example, the low baseline resistance rates to linezolid and bedaquiline support the implementation of regimens containing bedaquiline, pretomanid, and linezolid in China.^21^ Combination use of NRDs should be carefully formulated to prevent the development of co-resistance, which could occur quickly with overuse of NRDs.^22^ Group 1 mutations associated with resistance in our dataset were included in the first edition of the WHO catalogue, but group 2 mutations (especially indel mutations) in our dataset were not included in the WHO catalogue. Our results verify the accuracy of the WHO catalogue and show unique drug resistance mutations in China. The main reason for this finding might be the misuse of antibiotics or non-standard treatment of tuberculosis leading to arising high selection pressure for drug-resistant mutations in China. The proportion of present as a solo of each mutation also shows complex and diverse drug resistance profiles in China, but as the samples in the WHO catalogue expand, we expect that they will cover more mutations. For example, an additional 25 mutations of group 2 from our dataset are included as groups 1 or 2 in the second edition of the WHO catalogue (appendix 2 p 9).^23^ The mutation *gyrA*_Ala288Asp associated with levofloxacin resistance was newly identified in our dataset. Although this mutation has also been found in previous literature,^24^ to our knowledge, this study is the first to show statistical significance of *gyrA*_Ala288Asp resistance to levofloxacin. Based on the comparison between the wild-type and Ala288Asp mutant structures, the mutation results in the formation of new interactions between Asp288 of *gyrA* and loop1 of *gyrB* (appendix 2 p 8). This interaction might influence the drug entry mechanism, possibly obstructing levofloxacin from accessing the binding site. Some drug resistance mutations also showed differences in spatial distribution, such as *rpoB*_Ser450Leu and *gyrA*_Asp94Tyr, which might be caused by the spread of drug-resistant isolates or regional evolutionary pressure and therefore, the driving force of drug-resistant mutations might vary in different countries or regions.^25^

For mutations within group 1, we performed the Kruskal–Wallis test. There were statistically significant differences in the distribution of MIC values between the mutations associated with resistance to rifampicin, isoniazid, moxifloxacin, and levofloxacin. For example, the mutations at position 450 on *rpoB* showed high-level rifampicin resistance, and mutations at position 94 on *gyrA* showed high-level levofloxacin resistance. These results can facilitate the tailoring of individual drug doses based on molecular diagnostics. The mechanism behind differences in drug resistance levels needs to be further explained using protein–drug structure and molecular dynamics simulation.

We added two new indicators (the median of MIC and the FDR-corrected significance of the Wilcoxon rank-sum test) to measure the relationship between mutations and MIC values based on the WHO catalogue. Although some mutations were not in groups 1 and 2, the MICs of isolates with solo mutations were significantly higher than those of isolates without solo mutations, such as *ndh*_Val18Ala, *mshA*_Ala187Thr, and *mshA*_Arg413Gln, which have been reported in literature related to isoniazid resistance.^26^^,^^27^ Although the correlation between these mutations and drug resistance still needs to be studied,^28^ such mutations might still be associated with a moderate increase in MIC, which cannot be captured without phenotypic susceptibility testing.

The sensitivity and PPV of genetic-based predictions of resistance by our catalogue was low for ethambutol and ethionamide, which is most likely due to a combination of four factors. First, some drug-resistant mutations occur in combination and have a low frequency as solo mutations, so these were listed as mutations with uncertain significance in our catalogue (eg, *ahpC* promotor mutations measured by the WHO recommended rapid diagnostics Xpert MTB/XDR assay). These are probably compensatory mutations but they are good at predicting resistance. Second, although including a large number of isolates can help to overcome the variability in measurements, big data can still have systematic errors (eg, inaccuracies and changes in recommended critical concentrations).^11^^,^^29^ Third, epistatic effects exist in drug resistance mechanisms, especially in indel mutations, which might reverse the association with loss-of-function mutations.^30^ Fourth, most efflux pump genes and enzymes related to carbon source metabolism (such as *Rv2477c* and *glpK*) were not included in our two tiers of candidate genes, which might have led to missing some phenotypically-resistant isolates in the prediction.

Despite these limitations, as additional drug-resistant isolates are collected and drug-resistant genes are analysed, a more complete and accurate description of drug resistance loci in China becomes possible. Our analysis of drug-resistant isolates in China suggests that the association between indel mutations, efflux pump genes, protein structure, and MICs should be fully considered in the WHO catalogue and verified in future research, especially in countries with a high tuberculosis burden. With the generation of more data and improvements in analysis, a more comprehensive diagnosis and treatment of drug-resistant tuberculosis can be facilitated.

## Data sharing

All phenotypic data used in this study are shown in this manuscript and the appendix. The raw whole-genome sequencing data reported in this Article have been deposited in the Genome Sequence Archive (GSA: CRA017099) in the National Genomics Data Center, which is part of the China National Center for Bioinformation (Beijing Institute of Genomics, Chinese Academy of Sciences, Beijing, China), and are available online (https://ngdc.cncb.ac.cn/gsa).

## Declaration of interests

We declare no competing interests.
