# Supplementary material for: The catalogue of Mycobacterium tuberculosis mutations associated with drug resistance to 12 drugs in China from a nationwide survey: a genomic analysis
Source: Lancet Microbe. 2024 Nov;5(11):None. doi: 10.1016/S2666-5247(24)00131-9 (PMC11543636; doi:10.1016/S2666-5247(24)00131-9)
Supplement: Supplementary appendix 1 [file mmc1.pdf]

THE LANCET
Microbe

Supplementary appendix 1

This translation in Chinese was submitted by the authors and we reproduce it as supplied. It has not been peer reviewed. *The Lancet*'s editorial processes have only been applied to the original in English, which should serve as reference for this manuscript.

此简体中文译文由作者提交，我方按照提供的版本刊登。此译文并未经过同行审阅。医学期刊《柳叶刀》的编辑流程仅适用于英文原稿，英文原稿应作为此手稿的参考。

Supplement to: Pei S, Song Z, Yang W, et al. The catalogue of *Mycobacterium tuberculosis* mutations associated with drug resistance to 12 drugs in China from a nationwide survey: a genomic analysis. *Lancet Microbe* 2024. https://doi.org/10.1016/S2666-5247(24)00131-9

背景：2021 年世界卫生组织发布了第一版结核分枝杆菌耐药相关基因突变目录。然而，在不同国家，尤其是耐药高负担国家，可能会出现更为复杂和额外的耐药突变。本文旨在较为全面反映中国的耐药突变情况。

方法：我们分析了来自全国 31 个省、直辖市和自治区的 70 个县级结核病耐药监测点的结核分枝杆菌分离株。使用三种类型的药敏板对 12 种抗生素（利福平、异烟肼、乙胺丁醇、左氧氟沙星、莫西沙星、阿米卡星、卡那霉素、乙硫异烟胺、氯法齐明、利奈唑胺、德拉马尼和贝达喹啉）进行药敏试验。根据突变的阳性预测值 (PPV) 及其 95%置信区间、优势比(OR)和 FDR 校正后的 p 值，将突变分为五级：(1) 与耐药性相关；(2) 暂定与耐药性相关；(3) 不确定；(4) 暂定与耐药性无关；(5) 与耐药性无关。使用 Wilcoxon 秩和检验和 Kruskal-Wallis 检验量化突变与最低抑菌浓度 (MIC) 之间的关联。并将我们的数据集与世界卫生组织发布的第一版结核分枝杆菌耐药相关基因突变目录进行了比较。

结果：我们收集了 10 146 株结核分枝杆菌分离株，9071 株（89.4%）被纳入最终分析，其中，744 株（8.2%）对利福平耐药，1339 株（14.8%）对异烟肼耐药。在分析得到的 11,065 个突变中，208 个（1.9%）被归类为第一级和第二级。第 1 级中的 33 个（97.1%）和第 2 级中的 92 个（52.9%）突变也出现在 WHO 目录的第 1 级或第 2 级中。在第 2 级中的 81 个插入/缺失突变中，只有 15 个（18.5%）出现在 WHO 目录中。在我们数据集中新发现了一个与左氧氟沙星耐药性相关突变 gyrA_Ala288Asp。第 1 级中利福平、异烟肼、莫西沙星和左氧氟沙星的耐药突变对应的 MIC 值存在显著差异 ($p<0.0001$)，并检测到 12 个高水平耐药突变。在第 3 级中，我们还发现 61 个突变所对应的 MIC 值略高于其他敏感株。在 945 株表型耐药但基因型预测敏感的分离株中，有 433 株 (45.8%) 在至少一个外排泵基因发生了突变。

解释：我们的分析反映了中国耐药突变的复杂性，并建议在后续的目录中需充分考虑插入/缺失突变、外排泵基因、蛋白质结构以及 MIC 的变化，特别是在结核病负担高的国家。
