# Supplementary material for: The catalogue of Mycobacterium tuberculosis mutations associated with drug resistance to 12 drugs in China from a nationwide survey: a genomic analysis
Source: Lancet Microbe. 2024 Nov;5(11):None. doi: 10.1016/S2666-5247(24)00131-9 (PMC11543636; doi:10.1016/S2666-5247(24)00131-9)
Supplement: Supplementary appendix 2 [file mmc2.pdf]

THE LANCET
Microbe

Supplementary appendix 2

This appendix formed part of the original submission and has been peer reviewed.
We post it as supplied by the authors.

Supplement to: Pei S, Song Z, Yang W, et al. The catalogue of *Mycobacterium tuberculosis* mutations associated with drug resistance to 12 drugs in China from a nationwide survey: a genomic analysis. *Lancet Microbe* 2024. https://doi.org/10.1016/S2666-5247(24)00131-9

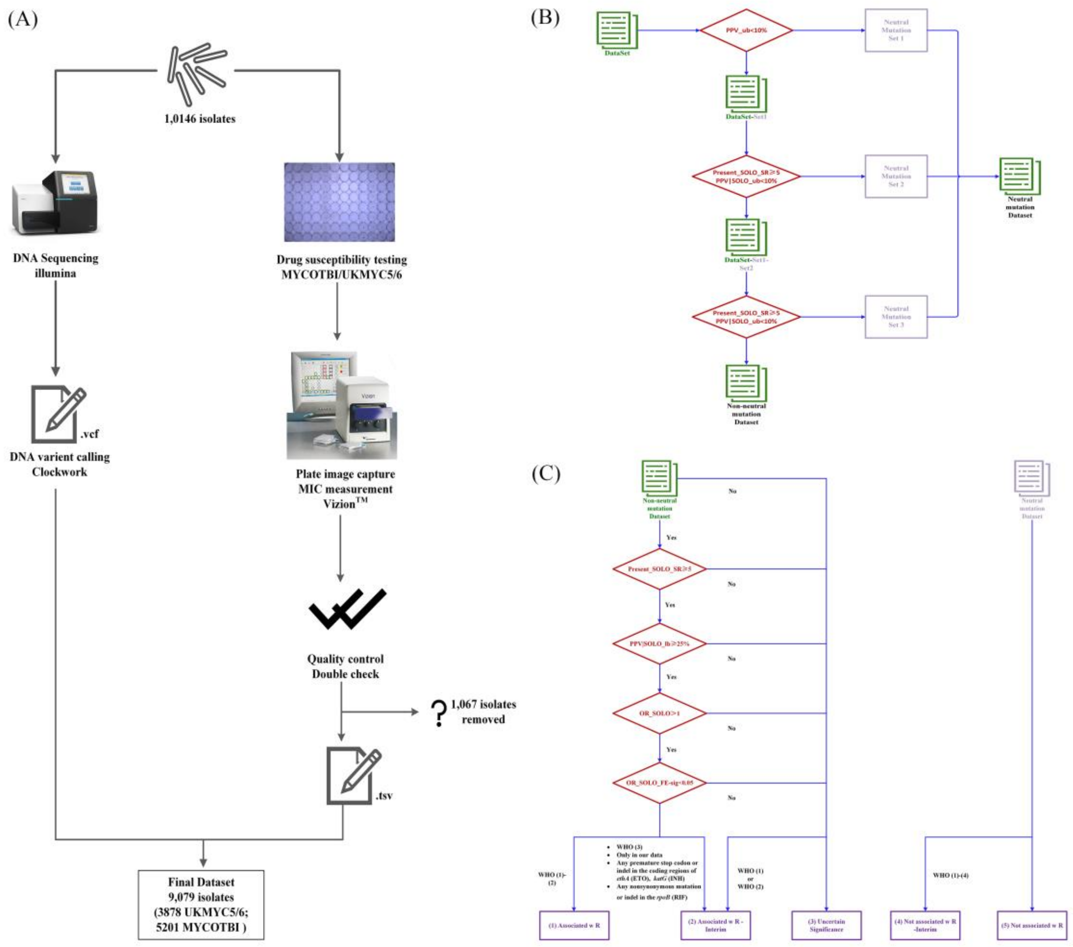

Figure S1: The overview of the processing of the data.
(A) Processing phenotypic and genotypic data for 10, 146 MTBC isolates. (B) Steps in the algorithm for determining neutral mutations. (C) Steps in the algorithm for the confidence grading.

Table S1: The candidate genes and critical concentration of 12 anti-tuberculosis drugs.

	**Tier 1**[a]	**Tier 2**[b]	**critical concentration used for interpretation (mg/L)**
**Rifampicin**	*rpoB*	*rpoA, rpoC, Rv1258c, Rv2752c*	0.5
**Isoniazid**	*ahpC, inhA, katG*	*mshA, ndh, Rv1258c, Rv2752c*	0.1[c]
**Moxifloxacin**	*gyrA, gyrB*	None	1

**Levofloxacin**	*gyrA, gyrB*	None	1
**Ethambutol**	*embA, embB, embC*	*embR, ubiA*	4
**Ethionamide**	*inhA, ethA*	*ethR, mshA, Rv3083, ndh*	4
**Kanamycin**	*rrs, eis, whiB7*	None	4
**Amikacin**	*rrs, eis, whiB7*	*whiB6, ccsA, fprA, aftB*	1
**Delamanid**	*Fgd1, ddn, fbiA, fbiB, fbiC, Rv2983*	None	0.125
**Clofazimine**	*pepQ, Rv0678, mmpL5, mmpS5*	*Rv1979c*	0.25
**Linezolid**	*rplC, rrl*	None	1
**Bedaquiline**	*pepQ, Rv0678, mmpL5, mmpS5, atpE*	None	0.25

The candidate genes were divided into two tiers according to their probability of containing resistance mutations.[1]

[a]Tier 1 comprised gene considered most probably to contain resistance mutations.

[b]Tier 2 included genes with a reasonable pre-test probability of containing resistance.

[c] Equivalent to the CLSI CC of 0.12 mg/L for the MYCOTB plate.[2]

Table S2: Linkage disequilibrium of drug resistant mutations

	*embB*_Met306Val	*embB*_Gln497Arg	*embB*_Tyr319Ser	*gyrA*_A Ala288Asp	*gyrA*_Ala90Val	*gyrA*_Asp94Ala	*gyrA*_Asp94Gly	*gyrA*_Asp94His	*gyrA*_Asp94Asn	*gyrA*_Asp94Tyr	*gyrA*_Ser91Pro	*gyrB*_Asp461Asn	*inhA*_C-777T	*inhA*_G-154A	*katG*_Ser315Asn	*katG*_Ser315Thr	*rpoB*_Asp435Val	*rpoB*_Asp435Tyr	*rpoB*_His445Asp	*rpoB*_His445Leu	*rpoB*_His445Tyr	*rpoB*_Leu452Pro	*rpoB*_Ser450Leu	*rpoB*_Ser450Trp	*rrs*_A1401G
*embB*_Met306Val	1.00	0.00	0.00	0.00	0.01	0.00	0.02	0.00	0.00	0.00	0.00	0.01	0.00	0.00	0.01	0.06	0.01	0.00	0.01	0.03	0.00	0.05	0.06	0.00	0.02
*embB*_Gln497Arg	0.00	1.00	0.00	0.00	0.00	0.00	0.01	0.00	0.00	0.00	0.00	0.00	0.00	0.00	0.00	0.01	0.02	0.00	0.00	0.00	0.00	0.00	0.02	0.02	0.00
*embB*_Tyr319Ser	0.00	0.00	1.00	0.00	0.00	0.00	0.02	0.00	0.00	0.00	0.00	0.00	0.00	0.00	0.00	0.00	0.00	0.00	0.00	0.00	0.00	0.00	0.00	0.00	0.00
*gyrA*_A Ala288Asp	0.00	0.00	0.00	1.00	0.00	0.00	0.00	0.00	0.00	0.04	0.00	0.00	0.00	0.00	0.00	0.00	0.00	0.00	0.00	0.00	0.01	0.00	0.00	0.00	0.00
*gyrA*_Ala90Val	0.01	0.00	0.00	0.00	1.00	0.01	0.01	0.00	0.00	0.00	0.00	0.00	0.00	0.00	0.00	0.01	0.00	0.00	0.00	0.00	0.00	0.01	0.01	0.00	0.01
*gyrA*_Asp94Ala	0.00	0.00	0.00	0.00	0.01	1.00	0.00	0.00	0.00	0.00	0.00	0.00	0.00	0.00	0.00	0.00	0.00	0.00	0.00	0.01	0.00	0.00	0.01	0.00	0.00
*gyrA*_Asp94Gly	0.02	0.01	0.02	0.00	0.01	0.00	1.00	0.00	0.00	0.00	0.00	0.00	0.00	0.00	0.00	0.02	0.00	0.00	0.00	0.00	0.00	0.00	0.03	0.00	0.00
*gyrA*_Asp94His	0.00	0.00	0.00	0.00	0.00	0.00	0.00	1.00	0.00	0.00	0.00	0.00	0.00	0.00	0.00	0.00	0.00	0.00	0.00	0.00	0.00	0.00	0.00	0.00	0.01
*gyrA*_Asp94Asn	0.00	0.00	0.00	0.00	0.00	0.00	0.00	0.00	1.00	0.00	0.00	0.00	0.00	0.00	0.00	0.00	0.01	0.00	0.00	0.00	0.00	0.00	0.00	0.00	0.00
*gyrA*_Asp94Tyr	0.00	0.00	0.00	0.04	0.00	0.00	0.00	0.00	0.00	1.00	0.00	0.00	0.00	0.00	0.00	0.01	0.00	0.00	0.00	0.00	0.08	0.00	0.00	0.00	0.01

*gyrA*_ Ser91Pro	0.00	0.00	0.00	0.00	0.00	0.00	0.00	0.00	0.00	0.00	1.00	0.00	0.00	0.00	0.00	0.00	0.00	0.00	0.00	0.00	0.00	0.00	0.00	0.00	0.00
*gyrB*_ Asp461Asn	0.01	0.00	0.00	0.00	0.00	0.00	0.00	0.00	0.00	0.00	0.00	1.00	0.00	0.00	0.00	0.00	0.00	0.00	0.04	0.00	0.00	0.02	0.00	0.00	0.00
*inhA*_C-777T	0.00	0.00	0.00	0.00	0.00	0.00	0.00	0.00	0.00	0.00	0.00	0.00	1.00	0.00	0.00	0.00	0.00	0.00	0.00	0.00	0.00	0.00	0.01	0.00	0.00
*inhA*_G-154A	0.00	0.00	0.00	0.00	0.00	0.00	0.00	0.00	0.00	0.00	0.00	0.00	0.00	1.00	0.00	0.00	0.00	0.00	0.00	0.00	0.00	0.00	0.00	0.00	0.01
*katG*_ Ser315Asn	0.01	0.00	0.00	0.00	0.00	0.00	0.00	0.00	0.00	0.00	0.00	0.00	0.00	0.00	1.00	0.00	0.04	0.02	0.00	0.00	0.01	0.01	0.00	0.01	0.00
*katG*_ Ser315Thr	0.06	0.01	0.00	0.00	0.01	0.00	0.02	0.00	0.00	0.01	0.00	0.00	0.00	0.00	0.00	1.00	0.01	0.00	0.01	0.01	0.02	0.02	**0.14**	0.00	0.01
*rpoB*_Asp435Val	0.01	0.02	0.00	0.00	0.00	0.00	0.00	0.00	0.01	0.00	0.00	0.00	0.00	0.00	0.04	0.01	1.00	0.05	0.00	0.00	0.02	0.00	0.00	0.00	0.01
*rpoB*_ Asp435Tyr	0.00	0.00	0.00	0.00	0.00	0.00	0.00	0.00	0.00	0.00	0.00	0.00	0.00	0.00	0.02	0.00	0.05	1.00	0.00	0.00	0.03	0.00	0.00	0.00	0.00
*rpoB*_ His445Asp	0.01	0.00	0.00	0.00	0.00	0.00	0.00	0.00	0.00	0.00	0.00	0.04	0.00	0.00	0.00	0.01	0.00	0.00	1.00	0.00	0.00	0.01	0.00	0.00	0.00
*rpoB*_ His445Leu	0.03	0.00	0.00	0.00	0.00	0.01	0.00	0.00	0.00	0.00	0.00	0.00	0.00	0.00	0.00	0.01	0.00	0.00	0.00	1.00	0.00	0.00	0.00	0.00	0.00
*rpoB*_ His445Tyr	0.00	0.00	0.00	0.01	0.00	0.00	0.00	0.00	0.00	0.08	0.00	0.00	0.00	0.00	0.01	0.02	0.02	0.03	0.00	0.00	1.00	0.00	0.00	0.00	0.00
*rpoB*_ Leu452Pro	0.05	0.00	0.00	0.00	0.01	0.00	0.00	0.00	0.00	0.00	0.00	0.02	0.00	0.00	0.01	0.02	0.00	0.00	0.01	0.00	0.00	1.00	0.00	0.00	0.00
*rpoB*_ Ser450Leu	0.06	0.02	0.00	0.00	0.01	0.01	0.03	0.00	0.00	0.00	0.00	0.00	0.01	0.00	0.00	0.14	0.00	0.00	0.00	0.00	0.00	0.00	1.00	0.00	0.01

*rpoB*_ Ser450Trp	0.00	0.02	0.00	0.00	0.00	0.00	0.00	0.00	0.00	0.00	0.00	0.00	0.00	0.00	0.01	0.00	0.00	0.00	0.00	0.00	0.00	0.00	0.00	1.00	0.01
*rrs*_A1401 G	0.02	0.00	0.00	0.00	0.01	0.00	0.00	0.01	0.00	0.01	0.00	0.00	0.00	0.01	0.00	0.01	0.01	0.00	0.00	0.00	0.00	0.00	0.01	0.01	1.00

Table S3: The diagnostic values for mutations of each drug in grade 1

Drug	Mutation	Present_ SOLO_SR[a]	PPV[b]	PPV_ub[c]	PPV_lb[d]	PPV| SOLO[e]	PPV| SOLO_ub[f]	PPV| SOLO_lb[g]	OR_SOLO[h]	OR_SOLO_sig[i]	MIC_SOLO_sig[j]
Isoniazid	*katG*_ Ser315Thr	513	0.95	0.97	0.94	0.94	0.96	0.91	191.99	1.48e-323	6.34E-282
	*inhA*_C-777T	127	0.93	0.96	0.89	0.88	0.93	0.81	70.5	3.41E-84	9.74E-45
	*katG*_ Ser315Asn	42	0.97	1.00	0.88	0.95	0.99	0.8	218.88	7.80E-32	1.25E-22
	*inhA*_G-154A	12	0.90	0.99	0.70	0.85	0.9	0.55	57.11	2.07E-08	1.48E-05
Rifampicin	*rpoB*_ Ser450Leu	39	0.97	0.99	0.95	0.79	0.89	0.64	381.56	1.42E-46	3.09E-28
	*rpoB*_ His445Tyr	34	0.94	0.98	0.85	0.89	0.97	0.75	Inf	3.08E-38	4.09E-24
	*rpoB*_ Leu452Pro	30	0.74	0.85	0.60	0.64	0.79	0.46	38.10	8.17E-20	1.29E-15
	*rpoB*_ His445Asp	25	0.89	0.97	0.74	0.85	0.96	0.65	84.52	1.76E-21	2.75E-14
	*rpoB*_ Asp435Val	13	0.89	0.98	0.72	0.81	0.96	0.54	74.19	6.06E-13	3.69E-09
	*rpoB*_ Asp435Tyr	8	0.94	0.98	0.85	0.89	0.97	0.75	Inf	3.08E-38	3.72E-06
	*rpoB*_ Ser450Trp	5	1.00	1.00	0.69	1.00	1.00	0.48	Inf	3.80E-06	3.61E-05
	*rpoB*_ His445Leu	6	0.93	1.00	0.66	0.83	1.00	0.36	56.13	2.08E-05	6.10E-05
Moxifloxacin	*gyrA*_ Asp94His	5	1.00	1.00	0.63	1.00	1.00	0.48	Inf	1.37E-07	3.58E-05
	*gyrA*_ Ser91Pro	17	0.96	1.00	0.79	0.94	1.00	0.73	Inf	3.09E-24	7.11E-14
	*gyrA*_ Asp94Gly	95	0.94	0.98	0.89	0.93	0.97	0.86	1516.01	1.06E-132	2.89E-68
	*gyrA*_ Asp94Asn	26	0.91	0.98	0.76	0.89	0.98	0.71	290.48	1.55E-31	8.12E-19
	*gyrA*_ Asp94Tyr	23	0.84	0.95	0.66	0.79	0.93	0.58	113.65	3.63E-23	3.54E-16
	*gyrA*_ Ala90Val	66	0.74	0.82	0.65	0.65	0.76	0.53	73.90	1.18E-53	1.60E-39
	*gyrA*_ Asp94Ala	32	0.65	0.79	0.49	0.58	0.74	0.41	45.88	6.51E-22	1.48E-22
Levofloxacin	*gyrA*_ Ala288Asp	5	1.00	1.00	0.63	1.00	1.00	0.48	Inf	8.84E-05	8.84E-05
	*gyrA*_ Asp94His	5	1.00	1.00	0.63	1.00	1.00	0.48	Inf	5.90E-05	5.87E-05

	*gyrA*_ Asp94Tyr	23	0.97	1.00	0.83	0.96	1.00	0.78	438.9	2.45E-16	2.39E-16
	*gyrA*_ Ser91Pro	17	0.96	1.00	0.79	0.94	1.00	0.73	Inf	1.08E-12	1.08E-12
	*gyrA*_ Asp94Gly	95	0.96	0.99	0.91	0.95	0.98	0.88	1183.70	2.43E-124	3.11E-66
	*gyrA*_ Asp94Ala	30	0.93	0.99	0.81	0.91	0.98	0.75	593.97	1.34E-12	2.53E-19
	*gyrB*_ Asp461Asn	9	0.86	0.98	0.57	0.8	0.97	0.44	153.72	2.62E-08	7.94E-07
	*gyrA*_ Asp94Asn	26	0.94	0.99	0.80	0.92	0.99	0.75	241.09	1.32E-27	1.93E-16
	*gyrA*_ Ala90Val	66	0.93	0.9	0.86	0.90	0.96	0.80	366.61	1.23E-76	4.97E-39
**Ethambutol**	*embB*_ Met306Val	62	0.86	0.92	0.78	0.78	0.87	0.67	863.35	6.89E-94	1.20E-49
	*embB*_ Gln497Arg	12	0.76	0.92	0.53	0.64	0.87	0.35	114.47	1.15E-12	2.90E-08
	*embB*_ Tyr319Ser	6	0.83	1.00	0.36	0.83	1.00	0.36	181.97	8.53E-08	1.99E-06
**Ethionamide**	*inhA*_C-777T	195	0.77	0.82	0.71	0.73	0.79	0.66	187.66	1.46E-193	8.96E-110
**Amikacin**	*rrs*_A1401G	37	0.88	0.95	0.76	0.84	0.94	0.68	522.31	1.88E-54	6.26E-23
**Kanamycin**	*rrs*_A1401G	39	0.88	0.95	0.76	0.85	0.94	0.70	114.00	4.23E-37	6.10E-22

[a] Present_SOLO_SR: Sum of resistant and susceptible isolates with the solo mutation.

[b] PPV: Positive predictive value of mutation.

[c] PPV_ub: Up-bound of PPV.

[d] PPV_lb: Low-bound of PPV.

[e] PPV|SOLO: Positive predictive value conditional on being solo.

[f] PPV|SOLO_ub: Up-bound of PPV|SOLO.

[g] PPV|SOLO_lb: Low-bound of PPV|SOLO.

[h] OR|SOLO: Odds ratio of solo mutation.

[i] OR_SOLO_sig: Fisher's exact test for the false discovery rate (FDR)-corrected P for the OR SOLO.

[j] MIC_SOLO_sig: Wilcoxon rank sum test for the false discovery rate (FDR)-corrected P for the distribution of MIC.

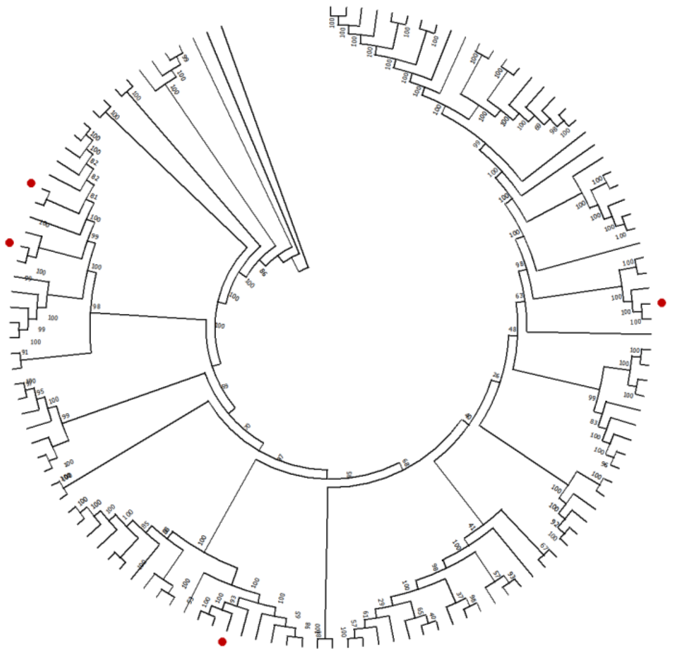

Figure S2: phylogenetic analysis of *gyrA*_ Ala288Asp. The red points represent the strains with the mutation "*gyrA*_ Ala288Asp".

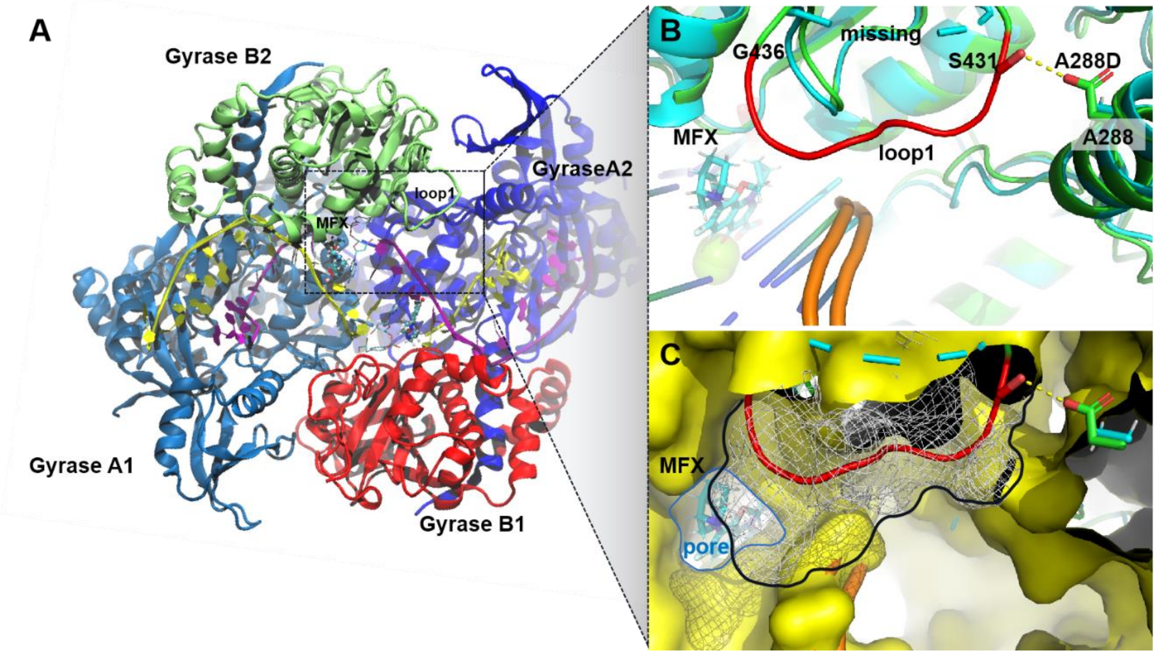

Figure S3: The modelled A288D structure formed hydrogen bond with S431.

(A). The overall structure of (*gyrA*+*gyrB*)dimer+DNA+MFX. (B). The carton comparison between X-ray structure WT (cyan) and A288D mutant (green). The modelled loop1(S431-G436) was colored in red. (C). The surface comparison between wt and Ala288Asp mutant, where the modelled loop was shown by white mesh.

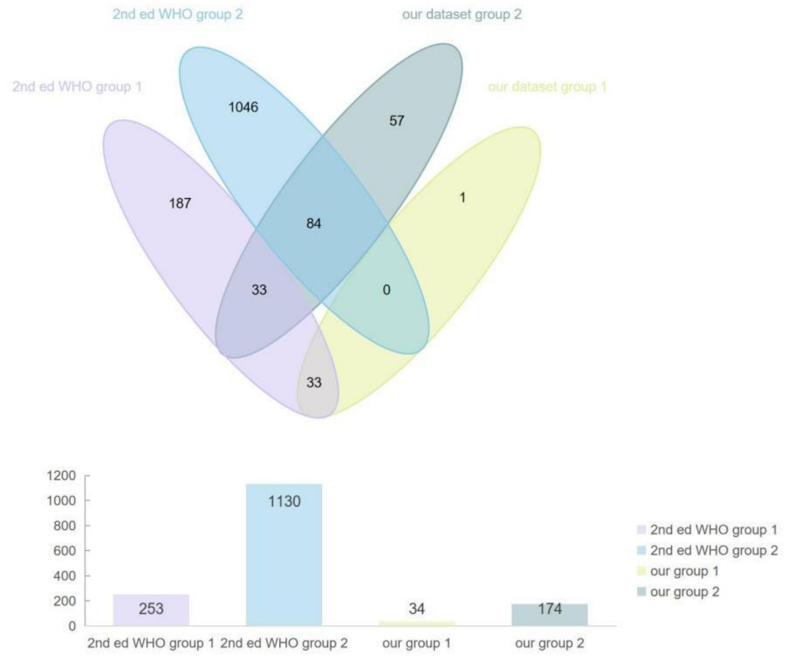

Figure S4: Venn diagram of the differences between our data and the second edition WHO catalogue for grade 1, 2 mutations.

Table S4: The correlation between northern and southern regions and drug-resistance mutations.

		**Northern region**	**Southern region**	**Total**	**Bonferroni-corrected p-value**
**Isoniazid**					
**Total**	*inhA*_C-777T	91	132	223	0.102
	*inhA*_G-154A	7	12	19	
	*katG*_ Ser315Asn	14	46	60	
	*katG*_ Ser315Thr	278	460	738	
**Lineage 2**	*inhA*_C-777T	70	92	162	0.114
	*inhA*_G-154A	5	8	13	
	*katG*_ Ser315Asn	9	31	40	
	*katG*_ Ser315Thr	250	361	611	
**Lineage 3**	*inhA*_C-777T	3	0	3	\
	*inhA*_G-154A	1	0	1	
	*katG*_ Ser315Asn	3	0	3	
	*katG*_ Ser315Thr	14	0	14	
**Lineage 4**	*inhA*_C-777T	18	39	57	0.021
	*inhA*_G-154A	1	4	5	
	*katG*_ Ser315Asn	2	15	17	
	*katG*_ Ser315Thr*	14	98	112	

**Rifampicin**					
**Total**	*rpoB_* Asp435Val	10	17	27	0.451
	*rpoB_* Asp435Tyr	5	9	14	
	*rpoB_* His445Asp	14	20	34	
	*rpoB_* His445Leu	4	10	14	
	*rpoB_* His445Tyr	28	39	67	
	*rpoB_* Leu452Pro	14	36	50	
	*rpoB_* Ser450Leu	150	207	357	
	*rpoB_* Ser450Trp	2	9	11	
**Lineage 2**	*rpoB_* Asp435Val	16	10	26	0.157
	*rpoB_* Asp435Tyr	8	5	13	
	*rpoB_* His445Asp	14	13	27	
	*rpoB_* His445Leu	10	3	13	
	*rpoB_* His445Tyr	26	27	53	
	*rpoB_* Leu452Pro	35	13	48	
	*rpoB_* Ser450Leu	162	137	299	
	*rpoB_* Ser450Trp	5	2	7	
**Lineage 3**	*rpoB_* Ser450Leu	0	1	1	\
	*rpoB_* Leu452Pro	0	4	4	
**Lineage 4**	*rpoB_* Asp435Val	1	0	1	0.34
	*rpoB_* Asp435Tyr	1	0	1	
	*rpoB_* His445Asp	6	1	7	
	*rpoB_* His445Leu	0	1	1	
	*rpoB_* His445Tyr	13	1	14	
	*rpoB_* Leu452Pro	1	0	1	
	*rpoB_* Ser450Leu	45	9	54	
	*rpoB_* Ser450Trp	4	0	4	
**EMB**					
**Total**	*embB*_M306V	32	81	113	0.870
	*embB*_Q497R	6	15	21	
	*embB*_Y319S	1	5	6	
**Lineage 2**	*embB*_M306V	25	74	99	1
	*embB*_Q497R	3	10	13	
	*embB*_Y319S	1	4	5	
**Lineage 3**	*embB_* Met306Val	2	2	4	1
	*embB_* Gln497Arg	1	3	4	
	*embB_* Tyr319Ser	0	0	0	
**Lineage 4**	*embB_* Met306Val	5	5	10	1
	*embB_* Gln497Arg	2	2	4	
	*embB_* Tyr319Ser	0	1	1	
**Fluoroquinolone**					
**Total**	*gyrA_* Ala288Asp	0	5	5	0.001
	*gyrA_* Ala90Val	25	74	99	

	*gyrA_* Asp94Ala	15	22	37	
	*gyrA_* Asp94Gly	30	83	113	
	*gyrA_* Asp94His	2	5	7	
	*gyrA_* Asp94Asn	5	24	29	
	*gyrA_* Asp94Tyr *	18	13	31	
	*gyrA_* Ser91Pro	5	15	20	
	*gyrB_* Asp461Asn	3	8	11	
**Lineage 2**	*gyrA_* Ala288Asp	1	4	5	0.003
	*gyrA_* Ala90Val	21	59	80	
	*gyrA_* Asp94Ala	15	18	33	
	*gyrA_* Asp94Gly	27	61	88	
	*gyrA_* Asp94His	2	2	4	
	gyrA_* Asp94Asn	2	15	17	
	*gyrA_* Asp94Tyr *	18	8	26	
	*gyrA_* Ser91Pro	5	11	16	
	*gyrB_* Asp461Asn	3	6	9	
**Lineage 3**	*gyrA_* Asp94Gly	1	0	1	\
**Lineage 4**	*gyrA_* Ala90Val	4	15	19	0.564
	*gyrA_* Asp94Ala	0	4	4	
	*gyrA_* Asp94Gly	2	22	24	
	*gyrA_* Asp94His	0	3	3	
	*gyrA_* Asp94Asn	3	9	12	
	*gyrA_* Asp94Tyr	0	4	4	
	*gyrA_* Ser91Pro	0	4	4	
	*gyrB_* Asp461Asn	0	2	2	

Table S5: The correlation between eastern, central, western regions and drug-resistance mutations.

		Eastern region	Central region	Western region	Total	Bonferroni-corrected p-value
**Isoniazid**						
**Total**	*inhA_*C-777T	59	124	40	223	0.459
	*inhA_*G-154A	5	10	4	19	
	*katG_* Ser315Asn	11	35	14	60	
	*katG_* Ser315Thr	169	387	182	738	
**Lineage 2**	*inhA_*C-777T	44	96	22	162	0.243
	*inhA_*G-154A	4	6	3	13	
	*katG_* Ser315Asn	10	21	9	40	
	*katG_* Ser315Thr	141	327	143	611	
**Lineage 4**	*inhA_*C-777T	14	28	15	57	0.240
	*inhA_*G-154A	1	4	0	5	
	*katG_* Ser315Asn	1	14	2	17	
	*katG_* Ser315Thr	27	60	25	112	

**Rifampicin**						
**Total**	*rpoB_* Asp435Val	4	13	10	27	0.003
	*rpoB_* Asp435Tyr	0	8	6	14	
	*rpoB_* His445Asp	6	20	8	34	
	*rpoB_* His445Leu	2	11	1	14	
	rpoB_* His445Tyr	21	30	16	67	
	*rpoB_* Leu452Pro	14	23	13	50	
	rpoB_* Ser450Leu	54	222	81	357	
	rpoB_* Ser450Trp	5	6	0	11	
**Lineage 2**	*rpoB_* Asp435Val	4	12	10	26	0.000
	*rpoB_* Asp435Tyr	0	8	5	13	
	*rpoB_* His445Asp	4	17	6	27	
	*rpoB_* His445Leu	2	11	0	13	
	rpoB_* His445Tyr	19	19	15	53	
	*rpoB_* Leu452Pro	14	22	12	48	
	rpoB_* Ser450Leu	44	194	61	299	
	*rpoB_* Ser450Trp	4	3	0	7	
**Lineage 4**	*rpoB_* Asp435Val	0	1	0	1	
	*rpoB_* Asp435Tyr	0	0	1	1	
	*rpoB_* His445Asp	2	3	2	7	
	*rpoB_* His445Leu	0	0	1	1	
	*rpoB_* His445Tyr	2	11	1	14	
	*rpoB_* Leu452Pro	0	1	0	1	
	*rpoB_* Ser450Leu	10	28	16	54	
	*rpoB_* Ser450Trp	1	3	0	4	
**EMB**						
**Total**	*embB_* Met306Val	23	68	22	113	0.108
	*embB_* Gln497Arg	1	17	3	21	
	*embB_* Tyr319Ser	0	6	0	6	
**Lineage 2**	*embB_* Met306Val	22	60	17	99	0.210
	*embB_* Gln497Arg	1	11	1	13	
	*embB_* Tyr319Ser	0	5	0	5	
**Lineage 4**	*embB_* Met306Val	1	4	5	10	0.720
	*embB_* Gln497Arg	0	3	1	4	
	*embB_* Tyr319Ser	01	1	0	1	
**Fluoroquinolone**						
**Total**	*gyrA_* Ala288Asp*	5	0	0	5	0.020
	*gyrA_* Ala90Val	20	64	15	99	
	*gyrA_* Asp94Ala	12	22	3	37	
	*gyrA_* Asp94Gly	19	79	15	113	
	*gyrA_* Asp94His	2	3	2	7	
	*gyrA_* Asp94Asn	4	18	7	29	

	*gyrA*_ Asp94Tyr *	16	11	4	31	
	*gyrA*_ Ser91Pro	3	15	2	20	
	*gyrB*_ Asp461Asn*	4	3	4	11	
**Lineage 2**	*gyrA*_ Ala288Asp*	4	1	0	5	0.000
	*gyrA*_ Ala90Val	13	54	13	80	
	*gyrA*_ Asp94Ala	11	19	3	33	
	*gyrA*_ Asp94Gly	17	63	8	88	
	*gyrA*_ Asp94His	2	2	0	4	
	*gyrA*_ Asp94Asn	2	13	2	17	
	*gyrA*_ Asp94Tyr *	16	7	3	26	
	*gyrA*_ Ser91Pro	2	12	2	16	
	*gyrB*_ Asp461Asn*	3	2	4	9	
**Lineage 4**	*gyrA*_ Ala90Val	7	10	2	19	0.253
	*gyrA*_ Asp94Ala	1	3	0	4	
	*gyrA*_ Asp94Gly	2	16	6	24	
	*gyrA*_ Asp94His	0	3	1	4	
	*gyrA*_ Asp94Asn	2	5	5	12	
	*gyrA*_ Asp94Tyr	0	3	1	4	
	*gyrA*_ Ser91Pro	1	3	0	4	
	*gyrB*_ Asp461Asn	1	1	0	2	

Reference

[1] WHO. Catalogue of mutations in *Mycobacterium tuberculosis* complex and their association with drug resistance. 2021. https://www.who.int/publications/i/item/9789240028173 (accessed June 25, 2021).

[2] Performance standards for susceptibility testing of mycobacteria, Nocardia spp., and other aerobic actinomycetes (CLSI supplement M62). Wayne (PA): Clinical and Laboratory Standards Institute; 2018.
